# The CN-12: A Brief, Multidimensional Connection With Nature Instrument

**DOI:** 10.3389/fpsyg.2020.01566

**Published:** 2020-07-14

**Authors:** Melissa Anne Hatty, Liam David Graham Smith, Denise Goodwin, Felix Tinoziva Mavondo

**Affiliations:** ^1^BehaviourWorks Australia, Monash Sustainable Development Institute, Monash University, Clayton, VIC, Australia; ^2^Department of Marketing, Monash Business School, Monash University, Clayton, VIC, Australia

**Keywords:** connection with nature, pro-environmental behavior, conservation, sustainability, behavior change, multidimensional instrument

## Abstract

In recent decades, there has been increasing interest in (re)connecting people with nature to foster sustainability outcomes. There is a growing body of evidence suggesting a relationship between connection with nature and pro-environmental behaviors. Connection with nature has often been conceptualized as a unidimensional construct, and although recent evidence suggests that it is multidimensional, there is ongoing debate regarding the dimensions that make up connection with nature. Existing multidimensional connection with nature instruments capture similar dimensions, yet they are lengthy and may not have practical application in real-world contexts. This research sought to clarify the dimensions of connection with nature and to develop and validate an abbreviated yet multidimensional connection with nature instrument—the CN-12. Analyses of two large datasets revealed three dimensions of connection with nature—identity, experience, and philosophy. Results suggested that the CN-12 and its three dimensions are positively correlated with: (1) environmental and altruistic values; (2) time spent in nature; and (3) a range of pro-environmental behaviors. Results also suggested that the CN-12 and its three dimensions are stable over time and are positively correlated with two existing multidimensional connection with nature instruments, the Nature Relatedness (NR) Scale and Environmental Identity (EID) Scale. The utility of the CN-12 for exploring human connections with nature and the role of fostering connection with nature to increase engagement in pro-environmental behaviors are discussed.

## Introduction

In recent decades, there has been increasing interest in human–nature relationships and the links between connection with nature (CN) and pro-environmental behaviors (PEB; Restall and Conrad, 2015; Ives et al., 2017). Disconnection from nature has been implicated as a key factor in ongoing environmental destruction (Nisbet et al., 2009; Zylstra et al., 2014), with some arguing that a sense of connection to nature is a necessary precondition for caring for, and commitment toward, protecting the natural environment (Schultz, 2002b). Thus, (re)connecting people with nature is seen as a potentially viable means of addressing sustainability outcomes (Seppelt and Cumming, 2016; Ives et al., 2018).

Such propositions are increasingly supported in the literature. A growing body of evidence suggests that PEB, that is, behaviors that result in minimal negative environmental impact or that protect or enhance the natural environment (Steg and Vlek, 2009), are more likely to occur among people who are more connected with nature (e.g., Geng et al., 2015; Richardson et al., 2019; Navarro et al., 2020). Two recent meta-analyses by Mackay and Schmitt (2019) and Whitburn et al. (2019) reported moderate positive correlations between CN and PEB (*r* = 0.37 and *r* = 0.42, respectively), providing further evidence of the potential utility in enhancing CN as a means of increasing engagement in PEB.

Discussions of how humans perceive, relate to, and interact with nature have seen the development of a range of terminology and definitions, such that the literature has become “characterized by a plurality of disciplinary and conceptual perspectives, language, methods and research approaches” (Ives et al., 2017, p. 106). Terminology used to describe human connections with nature include human–nature connectedness (Ives et al., 2017, 2018), connectedness to nature (Mayer and Frantz, 2004), nature relatedness (Nisbet et al., 2009), ecological identity (Walton and Jones, 2018), inclusion with nature (Schultz, 2002b), love and care for nature (Perkins, 2010), emotional affinity toward nature (Kals et al., 1999), and environmental identity (Clayton, 2003). Despite the plurality of definitions and terminology, a number of key themes are evident across definitions of CN. Typically conceptualized as subjective and personal, CN relates to a sense of personal identity, encompassing a relationship between the self and the natural world that includes cognitions, emotions, and behavior.

Considering the array of definitions of CN-related constructs, it is unsurprising that a diversity of self-report instruments have been developed that purport to capture CN. A summary of key instruments appears in Table 1. Most CN instruments have been developed to capture CN as unidimensional construct, although there is variability in the manner in which this construct has been conceptualized. Schultz (2001, 2002b), for example, considers CN as a cognitive construct; thus, the Inclusion of Nature in Self assesses an individual’s beliefs about their relationship with nature. Mayer and Frantz (2004) and Perkins (2010), in contrast, considered CN from an affective viewpoint, with the Connectedness to Nature Scale (CNS) and Love and Care for Nature (LCN) scale, respectively, assessing CN as an emotional construct^1^. Others have considered CN from a relational perspective, reflected in instruments such as the Connectivity with Nature scale (Dutcher et al., 2007) and Commitment to Nature scale (Davis et al., 2009). Interestingly, there have been no self-report instruments developed to date that consider CN as a purely experiential or behavioral construct. Some researchers have manipulated exposure to nature in experimental studies, for example, by watching a nature documentary (Zelenski et al., 2015; Arendt and Matthes, 2016); viewing pictures of, or walking in, nature (Klein and Hilbig, 2018; Nisbet et al., 2019; Mena-García et al., 2020); and multisensory nature experience via virtual reality (Soliman et al., 2017). Evidence suggests that exposure to nature may be related to the development of CN (Schultz and Tabanico, 2007; Braun and Dierkes, 2017; Cleary et al., 2018; Martin et al., 2020), although it is unclear whether exposure to nature is in fact an accurate representation of experiential or behavioral CN.

**TABLE 1:** Summary of key self-report connection with nature instruments, in chronological order.

Instrument	Author(s)	Dimensionality	Primary CN dimension(s) captured
Emotional Affinity Toward Nature (EATN)	Kals et al. (1999)	Unidimensional	Emotional
Inclusion of Nature in Self (INS)	Schultz (2001, 2002b)	Unidimensional	Cognitive
Environmental identity (EID)	Clayton (2003)	Multidimensional^2^	Cognitive
			Emotional
			Experiential
			Relationship
Connectedness to Nature Scale (CNS)	Mayer and Frantz (2004)	Unidimensional	Emotional^1^
Connectivity with Nature (CwN)	Dutcher et al. (2007)	Unidimensional	Relationship
Commitment to Nature (COM)	Davis et al. (2009)	Unidimensional	Relationship
Nature Relatedness (NR)	Nisbet et al. (2009)	Multidimensional	Cognitive
			Emotional
			Experiential
Love and Care for Nature (LCN)	Perkins (2010)	Unidimensional	Emotional
Disposition to Connect with Nature (DCN)	Brügger et al. (2011)	Multidimensional	Cognitive
			Emotional
			Experiential
Environmental connectedness (EC)	Beery (2013)	Unidimensional	Emotional
Nature Relatedness short form (NR6)	Nisbet and Zelenski (2013)	Unidimensional	Cognitive
Nature Connection Index (NCI)	Hunt et al. (2017), Richardson et al. (2019)	Unidimensional	Emotional
Ecological Identity Scale (EIS)	Walton and Jones (2018)	Unidimensional	Cognitive

Given the similarity between constructs, Tam (2013) empirically reviewed seven commonly cited CN instruments, with results suggesting a great degree of convergence between them. Tam’s findings suggested that multidimensional CN instruments performed better than unidimensional instruments, with the Environmental Identity (EID: Clayton, 2003) and Nature Relatedness (NR: Nisbet et al., 2009) scales showing consistently stronger correlations with criterion variables, including PEB, than unidimensional scales. Tam (2013) argued that “there are multiple aspects or dimensions of connection to nature, each of which has its own unique conceptual meanings but at the same time shares a substantial overlap with other aspects that warrants an identification of a common core” (p. 74). Thus, although instruments appear to be tapping a single underlying CN construct, different instruments emphasize different dimensions of CN (Tam, 2013).

Such findings are supported by two recent reviews. In a meta-analysis of studies exploring the relationship between CN and PEB, Whitburn et al. (2019) noted that the CN instrument used moderated the strength of the relationship between CN and PEB, with multidimensional CN scales, such as the EID, NR, and Disposition to Connect with Nature scale (DCN: Brügger et al., 2011) having the strongest relationships with PEB (*r* = 0.44, *r* = 0.51, and *r* = 0.53, respectively). Further, the authors classified each of the CN instruments as capturing (one or more of) affective, cognitive, or behavioral dimensions of CN, with results also suggesting that the dimensions captured moderated the relationship between CN and PEB. In a similar meta-analysis, Mackay and Schmitt (2019) reported that studies using the EID showed the strongest correlation between CN and PEB (*r* = 0.47), although studies using the multidimensional NR (*r* = 0.41) and unidimensional measures such as the CNS (*r* = 0.41) and emotional measures (e.g., the LCN: *r* = 0.44) showed similar correlations between CN and PEB. Together, these findings suggest that multidimensional CN instruments that distinguish between cognitive, emotional, and behavioral dimensions may be of greater utility in predicting engagement in PEB (Whitburn et al., 2019). Thus, further exploration of multidimensional CN instruments is warranted (Restall and Conrad, 2015).

In considering what dimensions, or combination of dimensions, best represent the CN construct, it is worth noting that definitions of CN typically include a sense of personal identity, encompassing a relationship between the self and the natural world that includes cognitions, emotions, and behavior. Therefore, these dimensions are clear potential candidates. Such ideas are reflected in the recent work of Ives et al. (2017, 2018) who conceptualize CN to comprise five distinct yet interrelated dimensions, or types of CN: philosophical, emotional, cognitive, experiential, and material. The authors consider these different types of CN to exist on a continuum from internal connections, such as worldviews about, and emotions associated with, nature (philosophical, emotional) to external connections, such as physical interaction with nature (material, experiential); these five CN dimensions are also considered relative to the scale of analysis, that is, at the individual and/or societal levels (Ives et al., 2018). These five dimensions are represented, to varying degrees, in two existing multidimensional CN instruments, with the exception of the material dimension (Table 2)^3^.

**TABLE 2:** Possible CN dimensions, captured by existing multidimensional CN instruments.

CN dimensions (Ives et al., 2017, 2018)	Environmental Identity (Clayton, 2003; Olivos and Aragonés, 2011)	Nature Relatedness (Nisbet et al., 2009)
*Philosophical*	*EID-Environmentalism*	*NR-Perspective*
Perspective or worldview on what nature is, why it matters, and how humans ought to interact with it (e.g., master, participant, steward); perspectives on humanity’s relationship to the natural world.	A perspective or ideology capturing commitment to, and behavior toward, the natural environment	A worldview; a sense of agency regarding human behavior and its impact on the natural environment
*Emotional*	*EID-Environmental identity*	*NR-Self*
Feelings of attachment to or empathy toward nature; emotional attachments and affective responses in relation to nature.	Self-identification and belonging represented by a sense of attachment or empathy, and thoughts about nature	An internal perspective or identity that includes emotions and thoughts about nature
*Cognitive*		
Knowledge or awareness of the environment and attitudes/values toward nature; knowledge, beliefs, and attitudes in relation to nature.		
*Experiential*	*EID-Enjoying nature*	*NR-Experience*
Direct interaction with natural environments (e.g., parks, forests); recreational activities in green environments.	Direct experience of nature and the pleasure associated with nature-based experiences	Desire to spend time in—and seeking out—nature, awareness of and fascination with nature
*Material*	*–*	*–*
Consumption of goods/materials from nature (e.g., food, fiber); resource extraction and use.		

As Table 2 shows, the EID and NR share three dimensions. The philosophical dimension, encompassing a worldview or ideology about nature including behaviors in relation to nature, is broadly captured by EID-Environmentalism and NR-Perspective. For Ives et al. (2018), this type of CN represents a person’s individual, internal connection yet may also represent the dominant worldview at a broader, societal scale. The experiential dimension, incorporating direct experiences of nature and enjoyment associated with such experiences, is broadly captured by EID-Enjoying nature and NR-Experience. This type of CN represents a more external connection via physical interactions with nature, typically analyzed at the individual level although can be aggregated to capture societal-level experiences (Ives et al., 2018). In their five-dimensional model, Ives et al. (2017, 2018) described distinct emotional and cognitive dimensions, representing internal connections at the individual level; yet the EID and NR appear to capture these dimensions under a single “identity” dimension (EID-Environmental identity and NR-Self). According to the identity theory, identities involve both cognitive and emotional processes (Stets and Biga, 2003; Burke and Stets, 2009), whereas evidence from psychology and cognitive neuroscience suggests that cognitions and emotions influence each other, such that distinguishing the two mechanisms may be difficult (Phelps, 2006; Barrett et al., 2007; Lerner et al., 2015). Thus, it seems prudent that the EID and NR capture cognitive and emotional dimensions under a single construct of identity.

Interestingly, although the EID and NR are considered multidimensional instruments, there have been few published studies that have explored the unique contribution of individual dimensions to PEB. In developing the NR, Nisbet et al. (2009), for example, reported that the NR-Self and NR-Perspective dimensions predicted vegetarianism whereas NR-Total and NR-Experience did not. Similarly, Forstmann and Sagioglou (2017) reported that only the NR-Self dimension predicted PEB. Indeed, Nisbet et al. (2009) noted that although the NR dimensions “sometimes showed different relationships with criterion variables, these differences were not overwhelming and never went in opposite directions … suggest[ing] that the factor structure requires further investigation” (p. 732). To the best of our knowledge, only two published studies have considered the unique contribution of EID dimensions to PEB, with EID-Environmentalism and EID-Environmental identity the strongest predictors of PEB (Olivos and Aragonés, 2011; Olivos et al., 2014). Taken together, these findings suggest that the dimensions comprising CN, and the potentially unique contribution of these CN dimensions to PEB, warrant further investigation.

Another issue with existing multidimensional CN instruments is in their length. The DCN, EID, and NR are relatively long instruments (40, 24, and 21 items, respectively), which may not be suitable for real-world contexts where time and money are limited (Maloney et al., 2011). A longer instrument also risks lower response rates and poorer data quality than a shorter instrument (Marcus et al., 2007; Galesic and Bosnjak, 2009). Although shorter versions of the EID and NR have been developed, these brief instruments are unidimensional in nature (Nisbet and Zelenski, 2013; Chew, 2019); thus, the potential utility and uniqueness of individual dimensions is lost. Therefore, there is utility in developing a multidimensional yet parsimonious CN instrument that can be used in real-world contexts.

The aims of the current research are threefold:

(1) to further explore and clarify CN dimensions, particularly relative to the five-dimensional model proposed by Ives et al. (2017, 2018);
(2) to develop a parsimonious instrument to capture a range of potential CN dimensions; and
(3) to assess the reliability, validity, and temporal stability of the CN instrument against criterion variables commonly used in CN research, including the extent to which specific CN dimensions may be related to different PEB.

Two studies were conducted to address these aims. Study 1 describes the analyses of an existing dataset, whereas Study 2 describes the collection and analyses of an additional dataset to complement and extend that described in Study 1.

## Study 1

Study 1 involved analyses of data (Hatty et al., 2018) presented in the report by Meis-Harris et al. (2019). This report proposed a new, 20-item multidimensional CN instrument, based loosely on the work of Ives et al. (2017, 2018) and intending to capture five CN dimensions: attachment (emotional), self (cognitive), materialism (material), experiential (experiential), and spirituality (philosophical). In the current research, data were analyzed to investigate the dimensionality of the CN instrument (Phase 1), to reduce the number of items while retaining a parsimonious, multidimensional instrument (Phase 2), and to assess construct validity against a series of criterion variables, including PEB (Phase 3). Ethics approval was granted by the Monash University Human Research Ethics Committee (Project ID: 14010).

### METHOD

#### Participants

Participants were recruited via on online panel survey company in exchange for a small financial reward. Participants under the age of 18 and those residing outside the Australian state of Victoria were excluded. The final sample (*N* = 3,090) was representative of residents in the state of Victoria with respect to age, gender, and geographical location (Meis-Harris et al., 2019).

#### Procedure and Questionnaire

Participants responded to a series of qualitative and quantitative questions assessing four broad areas. These included: (1) CN, 20 items intended to capture the five dimensions described above; (2) values, 12 items to capture biospheric (concern for the environment), altruistic (concern for people), and egocentric (concern for self) values; (3) engagement behaviors, five items capturing time spent in nature and beliefs about spending time in nature; and (4) PEB, 11 items capturing frequency of engaging in PEB in the past year (Meis-Harris et al., 2019). Items presented in blocks were randomized across participants to minimize question order effects. Data were collected in September and October 2018.

### Data Analyses and Results

All variables were screened for normality. Five of the CN items were skewed (item 9, −1.09; item 10, −0.78; item 16, −0.87; item 18, −1.39; and item 20, −1.11), however, transformations were not undertaken as doing so would make interpretation more difficult, and it was expected that the large sample would reduce the impact of non-normality on analyses (Tabachnick and Fidell, 2007).

We randomly split the total sample in two to facilitate analyses. Phase 1 involved exploratory factor analysis (EFA) using subsample 1 (*n* = 1,519). Phase 2 involved confirmatory factor analysis (CFA), based on the dimensions found in the EFA, conducted on subsample 2 (*n* = 1,571). Demographic characteristics for the two random samples were comparable with each other (Supplementary Table S1). Analyses were conducted using IBM SPSS Statistics Version 26 (IBM Corp., 2017), with CFA conducted using IBM SPSS AMOS Version 26 (Arbuckle, 2006).

#### Phase 1: Exploratory Factor Analysis

We conducted EFA on the 20 CN items to assess factor structure (*n* = 1,519). We used principal components analysis with promax rotation (κ = 4) as the goal was to explore the underlying component structure, and we expected the components to be correlated (Tabachnick and Fidell, 2007). Bartlett’s test of sphericity was significant (*p* < 0.001), and the Kaiser–Meyer–Olkin (KMO) measure was high (0.95), suggesting that the data were suitable for factor analysis. Communality values were between 0.54 and 0.75 for all items. A scree plot suggested a four component solution, accounting for 65.72% of the variance (Table 3). Factor loadings less than 0.3 are not shown (Hair et al., 2014).

**TABLE 3:** Exploratory factor analysis of the 20-item CN instrument (*n* = 1,519).

	Component			
	1	2	3	4
CN17. My connection to nature is something I would describe as “spiritual”	**0.92**			
CN1. I think of myself as an “environmentalist”	**0.75**			
CN4. My relationship to nature is a big part of how I think about myself	**0.68**			
CN19. Human beings and nature are connected by the same “energy” or “life-force”	0.67	−0.34	**0.51**	
CN3. Protecting nature is an important part of who I am	**0.61**			
CN8. I feel a strong emotional connection to nature	**0.60**	0.30		
CN5. I feel uneasy if I am away from nature for too long	**0.58**	0.35		
CN2. I think of myself as someone who is very concerned about taking care of nature	**0.51**			
CN10. I like to get outdoors whenever I get the chance		**0.82**		
CN9. I enjoy spending time in nature		**0.78**		
CN11. Being in nature allows me to do the things I like doing most		**0.67**		
CN12. Getting away on an overnight trip in nature is something I do as often as I can	0.30	**0.66**	−0.34	
CN6. I feel right at home when I am in nature		**0.65**		
CN7. Feeling connected to nature helps me deal with everyday stress	0.38	**0.43**		
CN18. Everything in nature is connected (e.g., animals, plants, humans, water, air, land, fire, etc.)			**0.81**	
CN20. Human wellbeing depends upon living in harmony with nature			**0.67**	
CN16. Natural areas are important to people because we use them for recreation	−0.36	0.45	**0.62**	
CN15. In order to provide us with the goods and services we need we can’t avoid nature being degraded.				**0.79**
CN13. Forests are valuable mostly because they produce wood products, jobs and income for people				**0.75**
CN14. Meeting the needs of people requires sacrificing some natural areas				**0.73**

The first component appears to represent an identity dimension with cognitive, emotional, and behavioral elements, including self-perception as someone who is emotionally connected to nature and who behaves in such a way as to protect nature. The second component represents an experiential dimension and includes activities undertaken in the natural environment. The third component represents a spiritual or philosophical dimension and embodies notions around humanity’s relationship with nature. The fourth component represents a materialism dimension and relates to notions around human use of natural resources.

A total CN score was calculated by averaging the 20 items, with scores for the four dimensions similarly calculated. Cronbach’s alpha for the 20-item CN scale and the four dimensions were calculated (Total, α = 0.90; Identity, α = 0.91; Experience, α = 0.88; Philosophy, α = 0.75; and Material, α = 0.66). Spearman correlations (with bias corrected and accelerated bootstrap 95% confidence intervals, shown in square brackets) indicated that the Identity dimension was strongly correlated with the Experience (*r*_s_ = 0.79, 95% BCa CI [0.77,0.81], *p* < 0.001) and Philosophy (*r*_s_ = 0.65, 95% BCa CI [0.61,0.68], *p* < 0.001) dimensions, and the Experience and Philosophy dimensions were strongly correlated (*r*_s_ = 0.60, 95% BCa CI [0.56,0.64], *p* < 0.001). The Material dimension was weakly and negatively correlated with the Identity dimension (*r*_s_ = −0.09, 95% BCa CI [−0.14, −0.03], *p* < 0.001), whereas correlations with the Experience (*r*_s_ = −0.03, 95% BCa CI [−0.08,0.03], *p* = 0.29) and Philosophy (*r*_s_ = −0.05, 95% BCa CI [0.10,0.00], *p* = 0.07) dimensions were non-significant.

#### Phase 2: Confirmatory Factor Analysis

We used CFA to verify the factor structure described in Phase 1 and to reduce the number of items to determine the most parsimonious model (Hair et al., 2014). We removed the materialism dimension first as this had the lowest internal consistency and the weakest and/or non-significant correlations with the other dimensions.

We inspected standardized factor loadings for individual items and removed items with loadings below 0.7; we also inspected modification indices and removed items with high cross-loadings (Hair et al., 2014). The standardized factor loading (regression weight) for item 19 was 0.677, thus below the “ideal” 0.7 cut-off point yet above the recommended minimum of 0.5; further, retaining item 19 ensured that the “philosophy” dimension contained the recommended minimum of three items (Hair et al., 2014).

**FIGURE 1:**
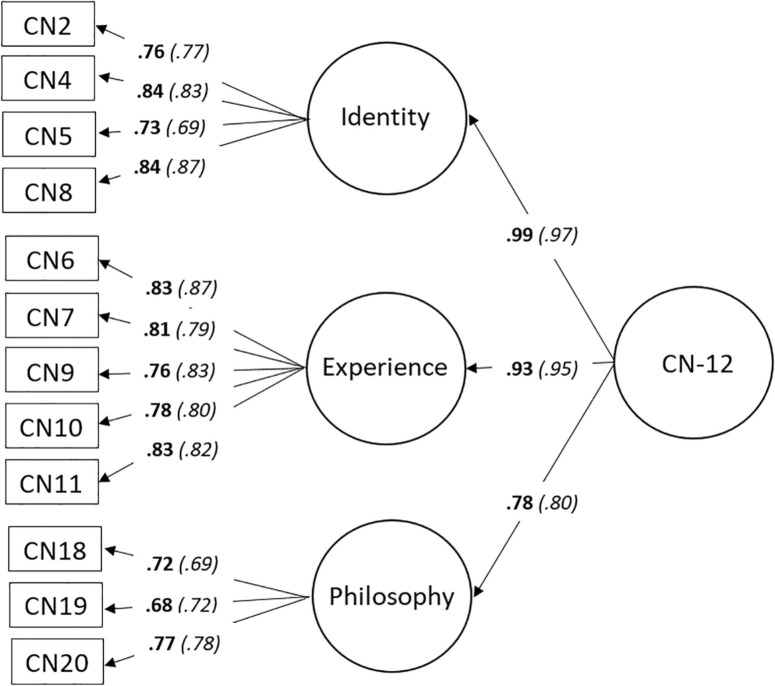
Measurement model for the CN-12. Study 1, *n* = 1,571; model fit indices: GFI = 0.95, AGFI = 0.92, NFI = 0.96, TLI = 0.95, CFI = 0.96, and RMSEA = 0.07; standardized regression weights shown in bold. Study 2, *n* = 526; model fit indices: GFI = 0.92, AGFI = 0.87, NFI = 0.94, TLI = 0.94, and CFI = 0.95; standardized regression weights shown italicized in brackets. GFI, goodness of fit index; AGFI, adjusted goodness of fit index; NFI, normed fit index; TLI, Tucker–Lewis index; CFI, comparative fit index; RMSEA, root mean square error of approximation.

The maximum likelihood method was used to test the second-order measurement model. A number of statistics were examined to assess the fit of the model. The goodness of fit index (GFI = 0.95), adjusted goodness of fit index (AGFI = 0.92), the normed fit index (NFI = 0.96), the Tucker–Lewis index (TLI = 0.95), the comparative fit index (CFI = 0.96), and the root mean square error of approximation (RMSEA = 0.07) suggested that the model was an acceptable-to-good fit of the data (Hu and Bentler, 1999; Schermelleh-Engel et al., 2003; Tabachnick and Fidell, 2007). A chi-square difference test indicated the Identity and Experience dimensions were distinct dimensions despite the high correlation between them (Hair et al., 2014). The 12-item model—the CN-12—is shown in Figure 1. Cronbach’s alpha for the CN-12 and three dimensions were calculated (CN-Total, α = 0.93; CN-Identity, α = 0.87; CN-Experience, α = 0.90; and CN-Philosophy, α = 0.75).

To confirm the three-dimensional structure of the CN-12, we conducted a second EFA (*n* = 1,571) using principal components analysis with promax rotation (κ = 4). A scree plot suggested a three component solution, accounting for 72.34% of the variance. The pattern and size of factor loadings were similar to those described above with the exception of item 7 loading more strongly on the identity dimension than the experience dimension and item 19 loading similarly on the identity and philosophy dimensions (Supplementary Table S2).

#### Phase 3: Validation and Relationships Between Connection With Nature and Pro-environmental Behavior

We used the total sample (*N* = 3,090) to validate the CN-12 via a series of Spearman correlations. We created an aggregate PEB score by calculating the mean of the 11 PEB. Consistent with previous research, we expected the CN-12 to be positively correlated with biospheric and altruistic values (Stern and Dietz, 1994; Schultz et al., 2005; Martin and Czellar, 2017), with time spent in nature (Rosa et al., 2018; Rosa and Collado, 2019), and with statements related to spending time in nature that capture general attitudes and beliefs about the natural environment (Cleary et al., 2018). We also expected the CN-12 to be positively correlated with the aggregate PEB score (Mackay and Schmitt, 2019; Whitburn et al., 2019), and with individual PEB (Perkins, 2010; Prévot et al., 2018a).

Results confirmed these hypotheses (Table 4). CN-12 scores (CN-Total, CN-Identity, CN-Experience, and CN-Philosophy) were positively related to biospheric and altruistic value orientations, to the amount of time spent in nature in the past year, and to beliefs about spending time in nature. Higher CN-12 scores were associated with greater frequency of participation in a range of PEB. CN-12 scores were weakly or non-significantly related to egoistic value orientation.

**TABLE 4:** Spearman correlations between the CN-12 total score, dimensions scores, and criterion variables (*N* = 3,090).

	CN-Total	CN-Identity	CN-Experience	CN-Philosophy
***Value orientations***				
Biospheric	0.68** [0.66,0.70]	0.62** [0.59,0.64]	0.59** [0.56,0.61]	0.67** [0.65,0.69]
Altruistic	0.46** [0.43,0.49]	0.39** [0.36,0.42]	0.40** [0.37,0.43]	0.50** [0.47,0.52]
Egoistic	0.02^ns^ [−0.02,0.05]	0.03^ns^ [−0.01,0.06]	0.03^ns^ [−0.01,0.06]	−0.08^ns^ [−0.07,0.00]
***Time spent in nature***				
In the past year	0.38** [0.35,0.41]	0.38** [0.34,0.41]	0.39** [0.37,0.42]	0.22** [0.19,0.25]
***Beliefs about time spent in nature***				
I spend as much time as possible in nature	0.64** [0.62,0.66]	0.61** [0.59,0.64]	0.66** [0.63,0.68]	0.40** [0.37,0.43]
It is important to me that my child/children spend time in nature (*n* = 723)^a^	0.53** [0.47,0.58]	0.43** [0.37,0.49]	0.53** [0.47,0.59]	0.45** [0.38,0.51]
I would like to spend more time in nature	0.53** [0.50,0.56]	0.46** [0.43,0.49]	0.53** [0.50,0.56]	0.41** [0.38,0.44]
***Pro-environmental behaviors (past year)***				
Aggregate PEB	0.46** [0.43,0.49]	0.50** [0.47,0.53]	0.39** [0.36,0.42]	0.32** [0.29,0.35]
Controlled the movements of pets (*n* = 1,556)^b^	0.25** [0.20,0.29]	0.23** [0.18,0.27]	0.21** [0.16,0.26]	0.24** [0.19,0.29]
Plant with native species	0.36** [0.33,0.39]	0.36** [0.33,0.40]	0.33** [0.30,0.36]	0.25** [0.22,0.28]
Reduced energy use	0.30** [0.27,0.33]	0.29** [0.26,0.32]	0.26** [0.22,0.29]	0.28** [0.25,0.31]
Chose sustainable seafood	0.33** [0.30,0.36]	0.34** [0.31,0.38]	0.27** [0.24,0.30]	0.26** [0.23,0.30]
Used public transport	0.07** [0.03,0.11]	0.08** [0.04,0.12]	0.06** [0.02,0.10]	0.04^∗^ [0.00,0.07]
Participated in environmental volunteering	0.29** [0.25,0.32]	0.33** [0.30,0.36]	0.25** [0.22,0.29]	0.16** [0.12,0.20]
Participated in citizen science	0.20** [0.16,0.23]	0.26** [0.22,0.29]	0.17** [0.14,0.20]	0.08** [0.04,0.11]
Donated to environmental organizations	0.33** [0.30,0.36]	0.36** [0.33,0.39]	0.26** [0.23,0.29]	0.25** [0.21,0.28]
Advocated for the environment	0.30** [0.26,0.32]	0.35** [0.32,0.38]	0.24** [0.21,0.27]	0.19** [0.16,0.23]
Cleaned up litter	0.34** [0.31,0.38]	0.36** [0.33,0.39]	0.31** [0.28,0.35]	0.22** [0.19,0.26]
Involved in community gardening or composting	0.19** [0.16,0.23]	0.25** [0.21,0.28]	0.16** [0.12,0.19]	0.09** [0.05,0.12]

Bias corrected and accelerated bootstrap 95% confidence intervals shown in brackets. ns, not significant (p > 0.05). ^a^Only shown to participants who reported they were the parent/guardian of a child/children aged 17 years or younger. ^b^Only shown to participants who reported owning a pet. *p < 0.05; **p < 0.01.

To determine the utility of individual dimensions in predicting individual PEB, we compared the correlations between CN-Total and PEB with correlations between each of the CN dimensions and PEB (Meng et al., 1992; Diedenhofen and Musch, 2015). Results suggested that the relationship between CN-Identity and PEB was significantly stronger than that between CN-Total and PEB for seven behaviors: chose sustainable seafood (*z* = −2.60, *p* = 0.009, 95% CI_diff_ [−0.032,−0.005]); participated in environmental volunteering (*z* = −6.92, *p* < 0.001, 95% CI_diff_ [−0.061,−0.034]); participated in citizen science (*z* = −8.96, *p* < 0.001, 95% CI_diff_ [−0.073,−0.047]); donated to environmental organizations (*z* = −5.88, *p* < 0.001, 95% CI_diff_ [−0.055,−0.027]); advocated for the environment (*z* = −8.74, *p* < 0.001, 95% CI_diff_ [−0.074,−0.047]); cleaned up litter (*z* = −2.29, *p* = 0.02, 95% CI_diff_ [−0.030,−0.002]); and involved in community gardening or composting (*z* = −8.16 *p* < 0.001, 95% CI_diff_ [−0.068,−0.042]). In contrast, the relationship between CN-Total and PEB was significantly stronger than that between CN-Identity and PEB for controlling the movement of pets (*z* = 2.35, *p* = 0.019, 95% CI_diff_ [0.004,0.039]).

## Study 2

Study 2 involved the collection of additional data (Hatty, 2019) to support and extend the analyses described in Study 1. In Phase 1, we used EFA and CFA to explore and confirm the dimensionality of the CN-12. In Phase 2, we used Spearman correlations to investigate the reliability, validity, and temporal stability of the CN-12 in relation to criterion variables (including PEB) and relative to two existing multidimensional CN instruments—the EID and NR. Ethics approval was granted by the Monash University Human Research Ethics Committee (Project ID: 21790).

### Method

#### Participants and Procedure

Participants who completed the survey described in Study 1 were re-contacted and invited to complete a follow-up survey. The survey was administered by the same online panel company and in a manner similar to that described in Study 1. Data were collected in September and October 2019. A total of 1,193 participants completed the survey, with 124 excluded from further analyses (52 did not consent to having responses from Study 1 and Study 2 matched; 21 could not have responses from Study 1 and Study 2 matched; and 51 provided conflicting information regarding age and/or gender between Study 1 and Study 2)^4^. The final sample for Study 2 (*N* = 1,069) comprised 48.7% females (*n* = 521) with age range of 19 to 88 years (*M* = 52.81, *SD* = 14.81). Owing to time and space limitations, demographic questions were limited to age and gender.

#### Questionnaire

A questionnaire was developed to validate the CN-12 described in Study 1, using items from the original questionnaire (the 20-item CN instrument, biospheric and altruistic value orientations, time spent in nature in the past year, and frequency of engaging in the 11 PEB in the past year). Two existing multidimensional CN instruments—the NR and EID—were included. Following feedback from pilot testing (*n* = 23), items were adapted to suit an Australian context (e.g., “forest” instead of “woods” and “holiday” instead of “vacation”) and amended to improve item clarity (e.g., “I would rather live in a small room or house with A NICE VIEW than a bigger room or house with a view of other buildings” was changed to “I would rather live in a small room or house with A VIEW OF NATURE than a bigger room or house with a view of other buildings”; “I really enjoy camping AND hiking outdoors” was changed to “I really enjoy camping AND/OR hiking outdoors”). Although the original NR instrument used a 5-point Likert scale, we used a 7-point scale to enable comparability with other CN instruments. A third multidimensional CN instrument—the DCN—was not included, as the inclusion of an additional 40 items would have added considerable time and cognitive burden.

### Data Analyses and Results

#### Phase 1: Exploratory and Confirmatory Factor Analysis

Analyses were conducted using IBM SPSS Statistics Version 26 (IBM Corp., 2017), with CFA conducted using IBM SPSS AMOS Version 26 (Arbuckle, 2006). Descriptive statistics provided an overview of the data, and variables were screened for normality. While five CN items were skewed (item 9, −1.11; item 10, 0.82; item 16, −0.84; item 18, −1.50; item 20, −1.21), transformations were not undertaken as doing so would make interpretation more difficult, and it was expected that the large sample would reduce the impact of non-normality on analyses (Tabachnick and Fidell, 2007).

We randomly split the database in two to facilitate validation of the CN-12. Using the first random sample (*n* = 543), we conducted EFA using principal components analysis with promax rotation (κ = 4). A scree plot suggested a three component solution, accounting for 74.08% of the variance. The pattern and size of factor loadings were consistent with those described in Study 1 with the exception of items 7 and 19 loading more strongly on the identity dimension than the experience and philosophy dimensions, respectively (Supplementary Table S3).

We conducted CFA (maximum likelihood) on the CN-12 using the second random sample (*n* = 526). The GFI (0.92), AGFI (0.87), NFI (0.94), TLI (0.94), and CFI (0.95) suggested the model was an acceptable-to-good fit of the data (Schermelleh-Engel et al., 2003; Tabachnick and Fidell, 2007; Figure 1).

Total CN was calculated by averaging the 12 items, with scores for the three CN dimensions calculated by averaging the items comprising each dimension (Figure 1). Cronbach’s alpha was calculated using the total sample (*N* = 1,069), with values for CN-Total (α = 0.94), and for the three dimensions (CN-Identity, α = 0.88; CN-Experience, α = 0.90; and CN-Philosophy, α = 0.77) consistent with Study 1. CN-Identity was strongly correlated with CN-Experience (*r*_s_ = 0.82, 95% BCa CI [0.79,0.84], *p* < 0.001) and CN-Philosophy (*r*_s_ = 0.64, 95% BCa CI [0.60,0.68], *p* < 0.001), and CN-Experience was strongly correlated with CN-Philosophy (*r*_s_ = 0.62, 95% BCa CI [0.58,0.67], *p* < 0.001).

#### Phase 2: Validation and Relationships Between Connection With Nature and Pro-environmental Behavior

As per Study 1, we used the total sample (*N* = 1,069) to assess construct validity of the CN-12. We calculated Spearman correlations between CN-Total, dimensions, and criterion variables including biospheric and altruistic value orientations, time spent in nature in the past year, and 11 PEB. Results were consistent with those from Study 1 (Supplementary Table S4).

To determine predictive validity of the CN-12, we calculated Spearman correlations between CN-Total and dimensions at Time 1 (Study 1: 2018) and criterion variables at Time 2 (Study 2: 2019). Correlations were consistent with those reported previously (Supplementary Table S5), indicating predictive validity.

To assess temporal stability of the CN-12, we calculated Spearman correlations (with bias corrected and accelerated bootstrap 95% confidence intervals shown in brackets) between scores at Time 1 (Study 1: 2018) and Time 2 (Study 2: 2019). Results suggested strong correlations between Time 1 and Time 2 for CN-Total (*r*_s_ = 0.77, 95% BCa CI [0.73,0.81], *p* < 0.001), CN-Identity (*r*_s_ = 0.75, 95% BCa CI [0.71,0.78], *p* < 0.001), CN-Experience (*r*_s_ = 0.72, 95% BCa CI [0.68,0.76], *p* < 0.001), and CN-Philosophy (*r*_s_ = 0.66, 95% BCa CI [0.63,0.70], *p* < 0.001). These results are consistent with those of prior research (Nisbet et al., 2011; Knepple Carney, 2018), indicating that CN is relatively stable over time.

As is common practice in the CN literature, we assessed convergent validity of the CN-12 using two existing CN instruments. In order to compare dimensions across instruments, we first explored the factor structure of the EID and NR using the total sample (*N* = 1,069). Owing to the lack of clarity around the dimensionality of the EID, we conducted EFA on the 24 items. A principal components analysis with promax rotation (κ = 4) revealed a four-component solution accounting for 61.51% of the variance (Supplementary Table S6). The factor structure was similar to that described by Olivos and Aragonés (2011), although the identity dimension included elements of the “environmentalism” dimension described by the authors. We conducted CFA (maximum likelihood) to verify the four-component model (Supplementary Table S7); fit indices suggested the model was a poor fit of the data (GFI = 0.87, AGFI = 0.84, NFI = 0.89, TLI = 0.89, CFI = 0.90, and RMSEA = 0.07), although removing item 7 improved the fit to an acceptable level (GFI = 0.90, AGFI = 0.87, NFI = 0.90, TLI = 0.91, CFI = 0.92, and RMSEA = 0.07) (Schermelleh-Engel et al., 2003; Tabachnick and Fidell, 2007). However, we retained item 7 in the final model to ensure consistency with previous literature.

The four dimensions were labeled EID-Identity, EID-Enjoying nature (experience); EID-Philosophy, and EID-Appreciation of nature. Cronbach’s alpha for the EID-Total and the four dimensions [EID-Total, α = 0.94; EID-Identity, α = 0.93; EID-Enjoying nature (experience), α = 0.79; EID-Philosophy, α = 0.83; EID-Appreciation of nature, α = 0.75] were comparable with those reported by Olivos and Aragonés (2011) (EID-Total, α = 0.90; EID-Environmental identity, α = 0.74; EID-Enjoying nature, α = 0.80; EID-Environmentalism, α = 0.80; EID-Appreciation of nature, α = 0.69). We calculated the total EID score by averaging all 24 items. We calculated scores for each of the four dimensions using the mean score of items in that dimension.

Prior to analyses of NR data, relevant items were reverse coded. As the factor structure of the NR requires further investigation (Nisbet et al., 2009), we conducted principal components analysis with promax rotation (κ = 4); a three-component solution was revealed, accounting for 55.70% of the variance (Supplementary Table S8). The factor structure was similar to that described by Nisbet et al. (2009), although with items 9, 19, and 20 loading on NR-Self and item 14 loading on NR-Perspective. We conducted CFA (maximum likelihood) to verify the three-component model (Supplementary Table S9); fit indices suggested the model was an adequate fit of the data (GFI = 0.88, AGFI = 0.85, NFI = 0.86, TLI = 0.86, CFI = 0.88, and RMSEA = 0.08) (Schermelleh-Engel et al., 2003; Tabachnick and Fidell, 2007). To ensure consistency with previous literature, we calculated mean scores for the NR-total and the three NR dimensions as per the authors’ guidelines (Nisbet et al., 2009). Cronbach’s alpha (NR-Total, α = 0.89; NR-Self, α = 0.87; NR-Experience, 0.76; NR-Perspective, α = 0.74) were consistent with those reported by Nisbet et al. (2009) (NR-Total, α = 0.87; NR-Self, α = 0.84; NR-Experience, 0.80; NR-Perspective, α = 0.66).

We calculated Spearman correlations between the CN-12, EID, and NR. We expected the total CN score to be positively correlated with total EID and NR scores. In considering the similar pattern of dimensions across the three instruments, we also expected the dimensions to correlate (CN-Identity with EID-Identity and NR-Self; CN-Experience with EID-Enjoying nature and NR-Experience; and CN-Philosophy with EID-Philosophy and NR-Perspective). Results confirmed these hypotheses (Table 5).

**TABLE 5:** Spearman correlations between the CN-12, Nature Relatedness Scale, and Environmental Identity Scale (total and dimension scores) (*N* = 1,069), with corresponding dimensions shown in bold (all correlations are statistically significant, *p* < 0.001).

	CN-Total	CN-Identity	CN-Experience	CN-Philosophy
NR-Total	**0.80 [0.78,0.83]**	0.75 [0.72,0.78]	0.73 [0.69,0.76]	0.68 [0.64,0.72]
NR-Self	0.83 [0.80,0.85]	**0.82 [0.80,0.84]**	0.72 [0.68,0.75]	0.68 [0.64,0.72]
NR-Experience	0.72 [0.68,0.75]	0.67 [0.63,0.71]	**0.74 [0.71,0.77]**	0.45 [0.40,0.50]
NR-Perspective	0.43 [0.38,0.49]	0.37 [0.31,0.43]	0.35 [0.29,0.40]	**0.52 [0.47,0.56]**
EID-Total	**0.82 [0.79,0.84]**	0.81 [0.78,0.83]	0.74 [0.71,0.77]	0.62 [0.57,0.66]
EID-Identity	0.75 [0.72,0.78]	**0.77 [0.74,0.80]**	0.64 [0.60,0.68]	0.59 [0.54,0.64]
EID-Enjoying nature (experience)	0.60 [0.55,0.65]	0.60 [0.55,0.65]	**0.62 [0.57,0.66]**	0.32 [0.26,0.37]
EID-Philosophy	0.75 [0.71,0.79]	0.68 [0.64,0.72]	0.71 [0.67,0.75]	**0.63 [0.59,0.68]**
EID-Appreciation of nature	0.65 [0.61,0.69]	0.65 [0.61,0.69]	0.58 [0.54,0.63]	0.50 [0.45,0.55]

*Bias corrected and accelerated bootstrap 95% confidence intervals shown in brackets.*

## Discussion

This research sought to: (1) further explore and clarify CN dimensions; (2) develop a parsimonious instrument to capture a range of potential CN dimensions; and (3) assess the reliability, validity, and temporal stability of the instrument against criterion variables commonly used in CN research, including PEB. Analyses of two large datasets revealed a 12-item CN instrument capturing three dimensions: Identity, Experience, and Philosophy. Results suggested that scores on the CN-12 (total and dimensions) are positively related to biospheric and altruistic values, time spent in nature, general attitudes toward spending time in nature, and 11 different PEB. Results also suggested that the CN-12 was stable over a 12-month period, with total and dimension scores strongly related to two existing multidimensional CN instruments.

### Connection With Nature Dimensions

In responding to calls for further exploration of the dimensionality of CN (Tam, 2013; Restall and Conrad, 2015), this research revealed three dimensions that broadly represent four of the five described by Ives et al. (2017, 2018). CN-Identity includes cognitive, emotional, and behavioral elements, including self-perception as someone who feels emotionally connected to nature and who behaves in such a way as to protect nature. CN-Experience represents a sense of enjoyment, wellbeing, and belonging associated with activities undertaken in the natural environment. The CN-Philosophy dimension embodies ideas around humanity’s relationship with nature, including a sense of interconnectedness between humans and nature. Together, these three dimensions align with existing definitions of CN as a sense of personal identity, encompassing a relationship between the self and the natural world that includes cognitions, emotions, and behavior. Two dimensions, CN-Experience and CN-Philosophy, are closely aligned with the experiential and philosophical dimensions (Ives et al., 2017, 2018). Although Ives et al. (2017, 2018) and Meis-Harris et al. (2019) proposed that emotional and cognitive CN are distinct, albeit related, dimensions, the results of the present studies suggest that these two dimensions can be aligned under the CN-Identity dimension. This is consistent with previous research suggesting that an identity dimension may broadly capture emotions and cognitions (Nisbet et al., 2009; Olivos and Aragonés, 2011). In addition, the dimensions captured by the CN-12 are conceptually similar to those of the NR and the EID. The moderate-to-strong correlations between the Identity (CN-Identity, EID-Identity, and NR-Self), Experience (CN-Experience, EID-Enjoying nature, and NR-Experience), and Philosophy (CN-Philosophy, EID-Philosophy, and NR-Perspective) dimensions suggest that the three instruments likely have a similar underlying structure. This provides further evidence that Identity, Experience, and Philosophy are important dimensions of the CN construct.

In the analyses presented above, CN-Identity accounted for the largest proportion of variance of the CN instrument. These results are similar to those described by Olivos and Aragonés (2011) and Nisbet et al. (2009), who noted that the EID-Environmental Identity and NR-Self dimensions, respectively, accounted for the largest proportion of variance. This suggests that identity may make the most significant contribution to the CN construct, relative to other dimensions. Interestingly, a recent meta-analysis suggested that CN and environmental identity were distinct yet highly correlated constructs (Balundė et al., 2019). However, 10 of the 11 studies included in the meta-analyses assessed environmental identity using the EID; thus, it is plausible that the EID-Identity dimension made the largest contribution to the overlap between environmental identity and CN in that study. Nevertheless, it appears that a sense of self-identification with the natural environment—encompassing thoughts, beliefs, and attitudes as well as emotional responses about and toward nature—is an integral part of CN.

Similarly, the results presented here suggest that the CN-12 is strongly correlated with both the EID and NR, in total and in dimension scores. Although this may suggest redundancy in the CN-12, it is worth noting that the three existing multidimensional CN instruments—the EID, NR, and the DCN—are lengthy, with 21, 24, and 40 items, respectively. The CN-12 is significantly shorter than existing multidimensional instruments while also capturing three dimensions; brief versions of the EID and NR, in contrast, are unidimensional (Nisbet and Zelenski, 2013; Chew, 2019). Preliminary evidence also suggests that different dimensions of the CN-12 may be stronger predictors of some PEB than other dimensions or the total CN score.

### Connection With Nature and Pro-environmental Behavior

Consistent with the findings of Nisbet et al. (2009), the results of these studies suggest some differences in the strength of correlations between CN dimensions and particular PEB. The relationships between CN-Identity and PEB were significantly stronger than the relationships between CN-Total and PEB for seven specific behaviors, including environmental volunteering, citizen science, donations to environmental organizations, and advocacy for the environment. From an applied perspective, fostering a sense of emotional connection to nature and self-identification as someone who protects nature (CN-Identity) may facilitate engagement in these seven behaviors.

The pattern and magnitude of correlations between the CN-12 and the aggregate PEB score, and between the CN-12 and individual PEB presented here were consistent with prior research (Prévot et al., 2018a; Mackay and Schmitt, 2019; Whitburn et al., 2019). This provides further evidence that people higher in CN tend to engage in a greater number of PEB and with greater frequency, than people lower in CN. Thus, fostering a sense of CN, and particularly CN-Identity, may be a useful means of encouraging engagement in PEB.

### Limitations

This paper details the development of a brief, multidimensional CN instrument with sound psychometric properties that is related to existing multidimensional CN instruments and to PEB. Nevertheless, a number of limitations are evident. Differences were noted in the strength of relationships between some CN dimensions and PEB, and although these were statistically significant, they were also relatively small. Although this provides preliminary evidence of the utility of individual CN dimensions in predicting specific PEB, further exploration of such relationships is warranted.

From a methodological perspective, participants completed the 20-item version of the instrument at Time 1 (2018) and Time 2 (2019), limiting the ability to demonstrate that the overlapping variance between the 12-item and 20-item instruments is sufficient (Smith et al., 2000). In addition, the CN-12 should be administered with additional, independent samples to confirm reliability and validity (Smith et al., 2000).

Finally, most CN research to date has been conducted in developed countries (Restall and Conrad, 2015; Ives et al., 2017), and the present research is no exception. Although the samples described were representative of the population of Victoria, which may facilitate generalization to the wider Australian or perhaps Western populations, the representation of respondents from diverse cultural and ethnic groups or Indigenous populations was not explicitly considered. Thus, the applicability of the CN construct to non-Western cultural groups and individuals in developing countries remains largely unexplored. Evidence suggests that values and beliefs about, and attitudes toward, the natural environment differ across cultural groups (e.g., Schultz, 2002a); thus, cross-cultural variability of the CN construct—at both individual and societal levels—warrants further investigation.

### Future Research

Given that research into the dimensionality of the CN construct is still in its infancy, further exploration of other possible dimensions is indicated. In particular, Ives et al. (2017, 2018) described material CN as the consumption of materials from nature (e.g., food and fiber) and resource extraction and use, such ideas that have largely been unexplored in the CN literature (Ives et al., 2017). Also of interest is the EID-Appreciation of nature dimension that encompasses elements of asthetic appreciation of nature. Evidence suggests that perceptions of the asthetic beauty of nature may be related to CN (Zhang et al., 2014; Lumber et al., 2017); thus, further exploration of the role of asthetic appreciation in CN is warranted. Another potential dimension that merits investigation is spatial or contextual CN; that is, the role that specific geographical locations may have in CN (Klaniecki et al., 2018; Giusti, 2019), perhaps leveraging insights from the place attachment literature (e.g., Gosling and Williams, 2010; Ramkissoon et al., 2013; Beery and Wolf-Watz, 2014). As noted by Balundė et al. (2019), a comprehensive understanding of the breadth of the CN construct may provide insights that enable targeted interventions to foster PEB.

Another area of consideration for future research is the conceptualization of CN as trait versus state. Some authors consider CN to be a trait-like construct that is relatively stable over time (Clayton, 2003; Mayer and Frantz, 2004; Nisbet et al., 2009; Martin et al., 2020), a notion that is supported by the present studies. Yet research suggests that CN may be more state-like, dependent upon seasons and weather patterns (Duffy and Verges, 2010; Nisbet et al., 2011) and able to be manipulated, for example, through exposure to natural environments (Mayer et al., 2009). Some have argued that while a single exposure to nature may increase state CN, a more enduring trait-like CN—likely to be developed with repeated experiences in nature—may be needed to trigger PEB (Zelenski et al., 2015; Clayton, 2017; Prévot et al., 2018b). Given that people higher in CN are more likely to engage in PEB, further understanding of CN as a state-like construct could enhance interventions aimed at increasing PEB, particularly among people lower in state CN.

## Conclusion

In recent years, an increasing interest in human connections with nature has resulted in a variety of definitions of CN, as well as instruments, to capture the construct. Although most instruments are unidimensional, recent evidence suggests that CN is multidimensional, although there is ongoing debate as to which dimensions make up the CN construct. Existing multidimensional CN instruments capture a similar array of dimensions, however, they are lengthy and may not be suitable for real-world contexts. The present studies describe the development of a brief CN instrument–the CN-12–that is multidimensional and is strongly related to existing multidimensional CN instruments. With an increasing body of evidence suggesting a relationship between CN and PEB, fostering a sense of connection with the natural world, and particularly a sense of identity relative to nature, may be a useful means through which to foster sustainability outcomes.

## Data Availability Statement

The datasets presented in this study can be found in online repositories. The names of the repository/repositories and accession number(s) can be found at: Open Science Framework (https://osf.io/py2ad/?view_only=4ef4a4545aa6462 6aa7baaea6d9811f2).

## Ethics Statement

The studies involving human participants were reviewed and approved by Monash University Human Research Ethics Committee (Project IDs: 14010 and 21790). The participants provided their written informed consent to participate in these studies.

## Author Contributions

All authors contributed to the study conception and design. MH prepared the materials, collected the data, and wrote the first draft of the manuscript. MH and FM analyzed the data. All authors commented on previous versions of the manuscript, read and approved the final manuscript.

## Conflict of Interest

The authors declare that the research was conducted in the absence of any commercial or financial relationships that could be construed as a potential conflict of interest.

## Footnotes

1 While Mayer and Frantz (2004) argued that the CNS captures emotional connection with nature, later research by Perrin and Benassi (2009) suggested that the CNS captures cognitive CN or an individual’s beliefs about their connection with nature.

2 While Clayton (2003) proposed the EID to be multidimensional, the author noted that preliminary data suggested the instrument may be unidimensional. Further, whereas Olivos and Aragonés (2011) found evidence of a multidimensional model, a later work by Chew (2019) suggested that the EID was unidimensional.

3 Brügger et al. (2011) did not explicitly describe CN dimensions they are intending to capture using the multidimensional Disposition to Connect with Nature, although personal preferences and attitudes imply cognitive CN, “bonding with nature” implies emotional and perhaps a relationship or philosophical CN, whereas behaviors imply experiential CN. As dimensions have not been made explicit by the authors, the DCN is not included in the analysis described in Table 2.

4 As Study 1 and Study 2 were conducted approximately 12 months apart, it was expected that age would increase by 1 year between Study 1 and Study 2. An age difference of up to 2 years was considered acceptable to account for human error.

## Abbreviations

CFA: Confirmatory factor analysis
CN: Connection with nature
EFA: Exploratory factor analysis
EID: Environmental Identity scale
NR: Nature Relatedness scale
PEB: Pro-environmental behavior.
