# Supplementary material for: The CN-12: A Brief, Multidimensional Connection With Nature Instrument
Source: Front Psychol. 2020 Jul 14;11:1566. doi: 10.3389/fpsyg.2020.01566 (PMC7372083; doi:10.3389/fpsyg.2020.01566)
Supplement: Supplementary file 1 [file Table_1.docx]

*S1: Study 1 Demographic characteristics for the two subsamples (Total N = 3090)*

|  | *Subsample 1*  *(n = 1519)* | | *Subsample 2*  *(n = 1571)* | |
| --- | --- | --- | --- | --- |
| Variable | *n* | *%* | *n* | *%* |
| Age |  |  |  |  |
| 18-29 | 271 | 17.8 | 324 | 20.6 |
| 30-39 | 273 | 18.0 | 295 | 18.8 |
| 40-49 | 272 | 17.9 | 264 | 16.8 |
| 50-59 | 289 | 19.0 | 264 | 16.8 |
| 60+ | 414 | 27.3 | 424 | 27.0 |
| Gender |  |  |  |  |
| Male | 751 | 49.4 | 786 | 50.0 |
| Female | 767 | 50.5 | 784 | 49.9 |
| Other | 1 | .1 | 1 | .1 |
| Victorian region |  |  |  |  |
| Metro | 1270 | 83.6 | 1310 | 83.4 |
| Inner regional | 201 | 13.2 | 225 | 14.3 |
| Outer regional | 45 | 3.0 | 34 | 2.2 |
| Not classified | 3 | .2 | 2 | .1 |
| Language spoken at home |  |  |  |  |
| Only English | 1323 | 87.1 | 1376 | 87.6 |
| Other | 173 | 11.4 | 177 | 11.3 |
| Prefer not to say | 23 | 1.5 | 18 | 1.1 |
| Employment status |  |  |  |  |
| Employed full time (30+ hours) | 678 | 44.6 | 726 | 46.2 |
| Employed part time (<30 hours) | 180 | 11.8 | 178 | 11.3 |
| Employed casually | 68 | 4.5 | 68 | 4.3 |
| Self-employed | 116 | 7.6 | 103 | 6.6 |
| Student only | 31 | 2.0 | 42 | 2.7 |
| Student + employment | 35 | 2.3 | 45 | 2.8 |
| Home duties / volunteer | 79 | 5.2 | 92 | 5.9 |
| Retired | 287 | 18.9 | 270 | 17.2 |
| Unemployed | 45 | 3.0 | 47 | 3.0 |
