# Supplementary material for: The CN-12: A Brief, Multidimensional Connection With Nature Instrument
Source: Front Psychol. 2020 Jul 14;11:1566. doi: 10.3389/fpsyg.2020.01566 (PMC7372083; doi:10.3389/fpsyg.2020.01566)
Supplement: Supplementary file 2 [file Table_2.docx]

*S2: Study 1 Exploratory factor analysis of the CN-12, to confirm the three-dimensional structure (n = 1571).*

|  | Component | | |
| --- | --- | --- | --- |
|  | 1 | 2 | 3 |
| CN5: I feel uneasy if I am away from nature for too long | .92 |  |  |
| CN4: My relationship to nature is a big part of how I think about myself | .76 |  |  |
| CN8: I feel a strong emotional connection to nature | .71 |  |  |
| CN19: Human beings and nature are connected by the same ‘energy’ or ‘life-force’ | .59 | -.39 | .58 |
| CN7: Feeling connected to nature helps me deal with everyday stress | .58 | .31 |  |
| CN2: I think of myself as someone who is very concerned about taking care of nature | .45 |  |  |
| CN9: I enjoy spending time in nature |  | .87 |  |
| CN10: I like to get outdoors whenever I get the chance |  | .80 |  |
| CN6: I feel right at home when I am in nature | .32 | .59 |  |
| CN11: Being in nature allows me to do the things I like doing most | .48 | .53 |  |
| CN18: Everything in nature is connected (e.g. animals, plants, humans, water, air, land, fire, etc.) |  |  | .92 |
| CN20: Human wellbeing depends upon living in harmony with nature |  |  | .78 |
