# Supplementary material for: The CN-12: A Brief, Multidimensional Connection With Nature Instrument
Source: Front Psychol. 2020 Jul 14;11:1566. doi: 10.3389/fpsyg.2020.01566 (PMC7372083; doi:10.3389/fpsyg.2020.01566)
Supplement: Supplementary file 3 [file Table_3.docx]

*S3: Study 2 Exploratory factor analysis of the CN-12 (n = 543)*

|  | Component | | |
| --- | --- | --- | --- |
|  | 1 | 2 | 3 |
| CN10: I like to get outdoors whenever I get the chance | .87 |  |  |
| CN9: I enjoy spending time in nature | .81 |  |  |
| CN11: Being in nature allows me to do the things I like doing most | .78 |  |  |
| CN6: I feel right at home when I am in nature | .73 |  |  |
| CN19: Human beings and nature are connected by the same ‘energy’ or ‘life-force’ | -.37 | .85 | .33 |
| CN5: I feel uneasy if I am away from nature for too long | .30 | .72 |  |
| CN4: My relationship to nature is a big part of how I think about myself |  | .70 |  |
| CN8: I feel a strong emotional connection to nature |  | .67 |  |
| CN7: Feeling connected to nature helps me deal with everyday stress | .33 | .56 |  |
| CN2: I think of myself as someone who is very concerned about taking care of nature |  | .51 |  |
| CN18: Everything in nature is connected (e.g. animals, plants, humans, water, air, land, fire, etc.) |  |  | .90 |
| CN20: Human wellbeing depends upon living in harmony with nature |  |  | .76 |
