# Supplementary material for: The CN-12: A Brief, Multidimensional Connection With Nature Instrument
Source: Front Psychol. 2020 Jul 14;11:1566. doi: 10.3389/fpsyg.2020.01566 (PMC7372083; doi:10.3389/fpsyg.2020.01566)
Supplement: Supplementary file 4 [file Table_4.docx]

S4: Study 2 Correlations between the total CN, CN dimensions, and criterion variables

(N = 1069)

|  | CN-  Total | CN-Identity | CN-Experience | CN-Philosophy |
| --- | --- | --- | --- | --- |
| *Value orientations* |  |  |  |  |
| Biospheric | .68^***^  [.65, .72] | .61^***^  [.56, .65] | .60^***^  [.55, .64] | .69^***^  [.64, .72] |
| Altruistic | .51^***^  [.47, .56] | .44^***^  [.39, .48] | .45^***^  [.40, .49] | .55^***^  [.50, .59] |
| *Time spent in nature* |  |  |  |  |
| In the past year | .43^***^  [.38, .47] | .39^***^  [.34, .44] | .44^***^  [.39, .49] | .26^***^  [.21, .32] |
| *Pro-environmental behaviours (past year)* | | | | |
| Aggregate PEB | .46^***^  [.41, .51] | .50^***^  [.45, .55] | .41^***^  [.35, .46] | .28^***^  [.22, .34] |
| Controlled the movements of pets (*n* = 473)^a^ | .25^***^  [.16, .34] | .22^***^  [.13, .31] | .21^***^  [.12, .30] | .27^***^  [.18, .35] |
| Plant with native species | .40^***^  [.35, .45] | .40^***^  [.35, .45] | .39^***^  [.33, .44] | .26^***^  [.20, .31] |
| Reduced energy use | .34^***^  [.28, .39] | .32^***^  [.26, .38] | .30^***^  [.24, .36] | .30^***^  [.24, .35] |
| Chose sustainable seafood | .35^***^  [.30, .41] | .35^***^  [.29, .41] | .31^***^  [.25, .37] | .29^***^  [.23, .35] |
| Used public transport | .10^**^  [.03, .16] | .11^***^  [.04, .17] | .09^**^  [.03, .16] | .04 ^ns^  [-.03, .10] |
| Participated in environmental volunteering | .24^***^  [.18, .30] | .31^***^  [.25, .36] | .21^***^  [.15, .27] | .10^***^  [.04, .16] |
| Participated in citizen science | .24^***^  [.18, .30] | .31^***^  [.26, .36] | .22^***^  [.16, .28] | .07^*^  [.01, .13] |
| Donated to environmental organisations | .29^***^  [.24, .35] | .34^***^  [.29, .40] | .25^***^  [.19, .31] | .16^***^  [.10, .22] |
| Advocated for the environment | .32^***^  [.27, .38] | .39^***^  [.34, .44] | .27^***^  [.21, .32] | .18^***^  [.13, .24] |
| Cleaned up litter | .37^***^  [.32, .43] | .39^***^  [.33, .45] | .36^***^  [.31, .42] | .22^***^  [.16, .28] |
| Involved in community gardening or composting | .16^***^  [.10, .22] | .23^***^  [.17, .28] | .14^***^  [.08, .20] | .00 ^ns^  [-.06, .06] |

^a^ Only shown to participants who reported owning a pet

^*^ *p* < .05

^**^ *p* < .01

^***^ p < .001

^ns^ Not significant (*p >* .05)

Bias corrected and accelerated bootstrap 95% confidence intervals shown in brackets
