# Supplementary material for: The CN-12: A Brief, Multidimensional Connection With Nature Instrument
Source: Front Psychol. 2020 Jul 14;11:1566. doi: 10.3389/fpsyg.2020.01566 (PMC7372083; doi:10.3389/fpsyg.2020.01566)
Supplement: Supplementary file 5 [file Table_5.docx]

S5: Study 2 Correlations between the CN-Total and CN dimensions at Time 1 (Study 1: 2018)

and between criterion variables at Time 2 (Study 2: 2019) (N = 1069)

|  | Time 1 | | | |
| --- | --- | --- | --- | --- |
| Time 2 | CN-  Total | CN- Identity | CN-Experience | CN-Philosophy |
| *Value orientations* |  |  |  |  |
| Biospheric | .60^***^  [.55, .65] | .54^***^  [.49, .59] | .52^***^  [.46, .57] | .60^***^  [.55, .64] |
| Altruistic | .43^***^  [.38, .49] | .37^***^  [.31, .43] | .37^***^  [.31, .43] | .48^***^  [.43, .53] |
| *Time spent in nature* |  |  |  |  |
| In the past year | .38^***^  [.32, .43] | .36^***^  [.30, .41] | .41^***^  [.36, .46] | .21^***^  [.16, .27] |
| *Pro-environmental behaviours (past year)* | | | | |
| Aggregate PEB | .43^***^  [.38, .48] | .47^***^  [.42, .52] | .39^***^  [.34, .45] | .27^***^  [.21, .33] |
| Controlled the movements of pets (*n* = 473)^a^ | .23^***^  [.15, .32] | .20^***^  [.11, .29] | .21^***^  [.12, .30] | .22^***^  [.14, .30] |
| Plant with native species | .37^***^  [.31, .42] | .37^***^  [.32, .42] | .36^***^  [.31, .42] | .24^***^  [.18, .29] |
| Reduced energy use | .30^***^  [.24, .35] | .28^***^  [.22, .34] | .26^***^  [.21, .32] | .27^***^  [.22, .32] |
| Chose sustainable seafood | .31^***^  [.25, .36] | .32^***^  [.26, .37] | .26^***^  [.20, .32] | .24^***^  [.18, .29] |
| Used public transport | .09^**^  [.03, .14] | .09^**^  [.03, .15] | .07^*^  [.02, .13] | .04^ns^  [-.02, .11] |
| Participated in environmental volunteering | .23^***^  [.17, .28] | .28^***^  [.22, .33] | .20^***^  [.15, .26] | .10^**^  [.04, .16] |
| Participated in citizen science | .23^***^  [.17, .29] | .29^***^  [.24, .35] | .22^***^  [.16. .27] | .07^*^  [.01, .14] |
| Donated to environmental organisations | .30^***^  [.25, .36] | .34^***^  [.28, .40] | .26^***^  [.20, .32] | .19^***^  [.13, .25] |
| Advocated for the environment | .31^***^  [.25, .37] | .37^***^  [.32, .43] | .27^***^  [.21, .33] | .17^***^  [.12, .23] |
| Cleaned up litter | .35^***^  [.29, .41] | .35^***^  [.29, .41] | .35^***^  [.29, .40] | .22^***^  [.16, .27] |
| Involved in community gardening or composting | .17^***^  [.11, .24] | .22^***^  [.16, .28] | .16^***^  [.10, .22] | .04^ns^  [-.03, .10] |

^a^ Only shown to participants who reported owning a pet

^*^ *p* < .05

^**^ *p* < .01

^***^ p < .001

^ns^ Not significant (*p >* .05)

Bias corrected and accelerated bootstrap 95% confidence intervals shown in brackets
