# Supplementary material for: The CN-12: A Brief, Multidimensional Connection With Nature Instrument
Source: Front Psychol. 2020 Jul 14;11:1566. doi: 10.3389/fpsyg.2020.01566 (PMC7372083; doi:10.3389/fpsyg.2020.01566)
Supplement: Supplementary file 6 [file Table_6.docx]

*S6: Study 2 Exploratory factor analysis on the Environmental Identity scale (EID) (N = 1069)*

|  | Component | | | | |
| --- | --- | --- | --- | --- | --- |
|  | 1 | 2 | 3 | 4 |  |
| EID7: I have a lot in common with environmentalists as a group | .99 | -.35 |  |  |  |
| EID22: My own interests usually seem to coincide with the position advocated by environmentalists | .98 |  |  |  |  |
| EID2: Engaging in environmental behaviours is important to me | .76 |  |  |  |  |
| EID4: If I had enough time or money, I would certainly devote some of it to working for environmental causes | .70 |  |  |  |  |
| EID13: Behaving responsibly toward the Earth - living a sustainable lifestyle - is part of my moral code | .65 | .40 |  |  |  |
| EID11: Being a part of the ecosystem is an important part of who I am | .64 |  |  |  |  |
| EID9: I feel that I have a lot in common with other species | .52 |  | .36 |  |  |
| EID3: I think of myself as a part of nature, not separate from it | .46 | .33 |  |  |  |
| EID15: In general, being part of the natural world is an important part of my self-image | .34 |  |  |  |  |
| EID14: Learning about the natural world should be an important part of every child’s upbringing |  | .87 |  |  |  |
| EID19: I would feel that an important part of my life was missing if I was not able to get out and enjoy nature from time to time |  | .84 |  |  |  |
| EID16: I would rather live in a small room or house with a view of nature than a bigger room or house with a view of other buildings |  | .80 |  |  |  |
| EID21: I have never seen a work of art that is as beautiful as a work of nature, like a sunset or a mountain range |  | .73 |  |  |  |
| EID5: When I am upset or stressed, I can feel better by spending some time outdoors in nature |  | .60 |  |  |  |
| EID8: I believe that some of today’s social problems could be cured by returning to a more rural lifestyle in which people live in harmony with the land |  | .59 |  |  |  |
| EID24: I keep mementos from the outdoors, such as shells or rocks or feathers |  |  | .89 |  |  |
| EID12: I feel that I have roots to a particular geographic location that had a significant impact on my development |  |  | .67 |  |  |
| EID18: Sometimes I feel like parts of nature - certain trees, or storms, or mountains - have a personality of their own |  |  | .63 |  |  |
| EID23: I feel that I receive spiritual sustenance from experiences with nature | .31 |  | .60 |  |  |
| EID10: I like to garden |  |  | .42 |  |  |
| EID17: I really enjoy camping and/or hiking outdoors |  |  |  | .86 |  |
| EID20: I take pride in the fact that I could survive outdoors on my own for a few days |  |  |  | .75 |  |
| EID1: I spend a lot of time in natural settings |  |  |  | .68 |  |
| EID6: Living near wildlife is important to me; I would not want to live in a city all the time |  | .47 |  | .47 |  |
