# Supplementary material for: The CN-12: A Brief, Multidimensional Connection With Nature Instrument
Source: Front Psychol. 2020 Jul 14;11:1566. doi: 10.3389/fpsyg.2020.01566 (PMC7372083; doi:10.3389/fpsyg.2020.01566)
Supplement: Supplementary file 7 [file Table_7.docx]

*S7: Study 2 Confirmatory factor analysis on the Environmental Identity scale (EID) (N = 1069). Fit indices: GFI = .87, AGFI = .84, NFI = .89, TLI = .89, CFI = .90, RMSEA = .07.*

*
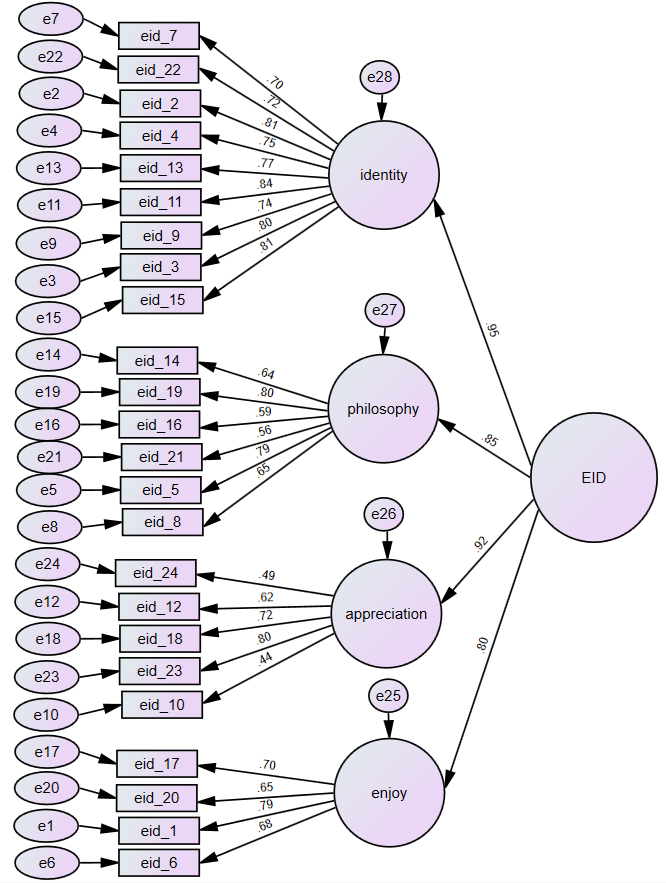
*
