# Supplementary material for: The CN-12: A Brief, Multidimensional Connection With Nature Instrument
Source: Front Psychol. 2020 Jul 14;11:1566. doi: 10.3389/fpsyg.2020.01566 (PMC7372083; doi:10.3389/fpsyg.2020.01566)
Supplement: Supplementary file 8 [file Table_8.docx]

*S8: Study 2 Exploratory factor analysis on the Nature Relatedness scale (NR) (N = 1069)*

|  | Component | | |
| --- | --- | --- | --- |
|  | 1 | 2 | 3 |
| NR5: I always think about how my actions affect the environment | .75 |  |  |
| NR7: My connection to nature and the environment is a part of my spirituality | .75 |  |  |
| NR8: I am very aware of environmental issues | .72 |  |  |
| NR9: I take notice of wildlife wherever I am | .68 |  |  |
| NR12: I am not separate from nature, but a part of nature | .74 |  |  |
| NR16: Even in the middle of the city, I notice nature around me | .54 |  |  |
| NR17: My relationship to nature is an important part of who I am | .73 |  |  |
| NR19: The state of nonhuman species is an indicator of the future for humans | .73 |  |  |
| NR20: I think a lot about the suffering of animals | .80 |  |  |
| NR21: I feel very connected to all living things and the earth | .82 |  |  |
| NR2: Some species are just meant to die out or become extinct (reverse scored) |  | .74 |  |
| NR3: Humans have the right to use natural resources any way we want (reverse scored) |  | .81 |  |
| NR11: Nothing I do will change problems in other places on the planet (reverse scored) |  | .65 |  |
| NR14: My feelings about nature do not affect how I live my life (reverse scored) |  | .59 |  |
| NR15: Animals, birds and plants have fewer rights than humans (reverse scored) |  | .48 |  |
| NR18: Conservation is unnecessary because nature is strong enough to recover from any human impact (reverse scored) |  | .80 |  |
| NR1: I enjoy being outdoors, even in unpleasant weather |  | .42 | .71 |
| NR4: My ideal holiday spot would be a remote, wilderness area |  |  | .53 |
| NR6: I enjoy digging in the earth and getting dirt on my hands |  |  | .46 |
| NR10: I don’t often go out in nature (reverse scored) |  |  | .70 |
| NR13: The thought of being deep in the forest, away from civilisation, is frightening (reverse scored) |  |  | .68 |
