# Supplementary material for: The CN-12: A Brief, Multidimensional Connection With Nature Instrument
Source: Front Psychol. 2020 Jul 14;11:1566. doi: 10.3389/fpsyg.2020.01566 (PMC7372083; doi:10.3389/fpsyg.2020.01566)

*S9: Study 2 Confirmatory factor analysis on the Nature Relatedness scale (NR) (N = 1069).*

*Fit indices: GFI = .88, AGFI = .85, NFI = .86, TLI = .86, CFI = .88, RMSEA = .08.*


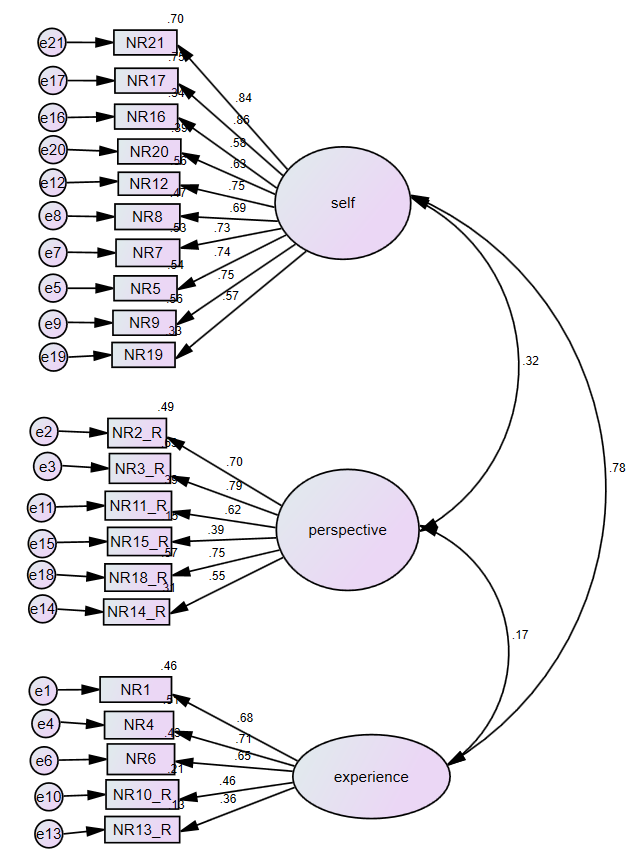

Supplement: Supplementary file 9 [file Table_9.docx]
